# Framework for the establishment of a feasible, tailored and effective perinatal education programme

**DOI:** 10.1186/s12884-017-1234-7

**Published:** 2017-02-08

**Authors:** Isabel Artieta-Pinedo, Carmen Paz-Pascual, Gonzalo Grandes, Maite Espinosa

**Affiliations:** 1 Primary Care Midwife Zuazo Health Centre, Barakaldo, (Bizkaia); and Associate Professor of the School of Nursing, University of the Basque Country, Leioa, Bizkaia, Spain; 2 Primary Care Midwife in Sestao Health Centre, Bizkaia; and Lecturer in the Midwifery Training Unit of the Basque Country, Bilbao, Bizkaia, Spain; 3 Primary Care Research Unit of Bizkaia, Centro de Salud de Deusto, 4ª planta, 48014 Bilbao, Bizkaia Spain

**Keywords:** Antenatal classes, Birth preparation, Perinatal education, Woman care, Prenatal care, Health literacy

## Abstract

**Background:**

Antenatal education needs to be renewed and adapted to the needs of women. Objectives: to assess women needs, identify factors that influence the desired outcomes, and propose a framework for developing new perinatal education based on the guidance published by the UK Medical Research Council for the development and evaluation of complex interventions in primary care.

**Methods:**

For this study: (a) four focus group sessions were held from October to November 2010 in Bizkaia (Spain) with 30 women exploring their needs during pregnancy and postpartum; (b) two literature reviews were conducted on women’s needs at these times and theoretical models of healthcare education; and (c) seven discussion and consensus sessions were run with a group of experts composed of midwifes, gynaecologists, paediatricians, and paediatric and postpartum nurses.

**Results:**

Various areas for improvement were identified: needs assessment of each woman/family, consideration of pregnancy and childbirth as normal physiological processes, participation of fathers, establishment of social networks, continuity of postpartum care, better access to and training for midwives, and more flexible format and contents for the programme. We propose a woman-focused framework that includes three exploratory interviews during pregnancy, personalized interventions coordinated between professionals, empowerment to choose the type of birth, and postpartum activities.

**Conclusion:**

New perinatal education should be on-going and focused on each woman. It is necessary to assess the feasibility of implementing this type of programme, depending on the context, professionals’ readiness for change and characteristics of the proposed interventions. Then, its effectiveness and sustainability must be assessed.

## Background

Research on antenatal education (AE) consistently indicates the need for adapting programmes to the needs of today’s women and making them more effective [1–3]. AE has remained relatively unchanged for the last 40 years while both childbirth and maternity have changed significantly. In highly medicalised settings, the potential benefits of AE are obscured by the actions of the health professional, [4] and it would appear to be more appropriate where childbirth is less-medicalised [5]. Despite this, AE is remarkably similar across all settings. The most common type of AE programme is composed of 8 to 10 sessions providing information and also teaching gym exercises, and breathing and relaxation techniques. The weight given to different parts of the content is influenced by the midwife running the sessions, and participation is voluntary. Often, little or no attention is paid in AE to the adoption of healthy habits that could prevent or ameliorate problems during the birth and postpartum, women’s autonomy during birth, or care of the infant, and as a result, the education tends not to improve the course of pregnancy or satisfaction among pregnant women [4, 6, 7], nor does it prolong breastfeeding as it should [8, 9]. Nevertheless, women demand this activity and participate in AE sessions, probably seeking guidance through the plethora of information available about childbirth, the sheer quantity hindering their decision-making [10]. This represents an opportunity for health professionals to promote the health of mothers and of new families, encouraging healthy lifestyles [11, 12], though it is not clear that current AE achieves this goal either [13]. Overall, it seems that AE is necessary [14], but that it needs to be redesigned to be more useful [15].

Redesigning AE is, however, a complex process. Following the model proposed by the Medical Research Council in the UK, our team is taking this process forward in five phases: a preclinical theoretical phase, modelling, piloting, randomized clinical trial(s), and long-term implementation of the intervention [16]. This article describes the preclinical phase, consisting of an analysis of the variables and strategies that may influence the desired results. To complete this phase, we have addressed the following specific objectives: (1) to identify theoretical models and strategies to make AE programmes more effective; (2) to evaluate these intervention strategies for health education; (3) to assess the care and health needs of women who are planning to get pregnant, are pregnant or have recently had a child and factors that affect these needs; (4) to identify areas for improvement in perinatal education regarding the processes of pregnancy, childbirth and child rearing; and (5) to reach a consensus among experts on the model, content and strategies of a new perinatal education programme focused on women’s needs that is feasible and potentially effective and efficient.

## Methods

**Fig. 1:**
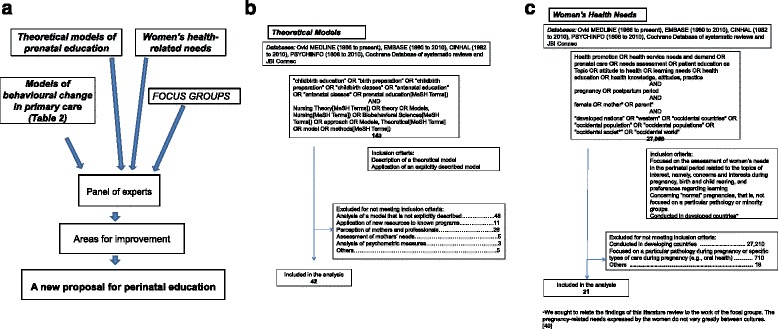
**a** Flow chart of the research process. **b** Search strategy used for maternal education models. **c** Search strategy used for women’s needs in maternal education

Formative research was carried out based on a literature review and qualitative research with focus groups [17]. The results were reported to a panel of experts on pregnancy, childbirth and the postpartum period that met for seven discussion and consensus sessions between May 2010 and November 2011 (Fig. 1a). A consensus methodology was employed because scientific evidence on which AE interventions are effective and factors determining their success is limited or lacking [6, 15, 18]. Further, an adapted nominal-group technique was used to promote debate, interaction and generation of a wide range of ideas. The target result was a proposal for AE which could be applied to produce improvements in maternal lifestyle and family health.

A first literature search was conducted in May 2010 focused on current patterns of AE. The process is illustrated in Fig. 1b. Further, we studied the models and theories about behaviour change for the promotion of healthy habits identified by Glanz et al. [19]. Among these, we selected examples of the application of these theories to pregnant women (toxic habits, weight gain, premature birth and low birth weight infants), postpartum period (inclusion of partner) or teenagers (contraceptive measures, and prevention of sexually transmitted diseases).

In parallel, a second literature search was performed to identify original articles and reviews concerning health needs in relation to pregnancy, birth and postpartum. The search strategy is shown in Fig. 1c. We selected original articles or systematic reviews of qualitative and quantitative descriptive studies whose central theme was the needs assessment of women during the perinatal period, conducted in developed countries, and published in English or Spanish between the 1998 and 2010. Papers concerning pathological conditions or minority groups were excluded (for example, those with a focus on human immunodeficiency virus, oral health or contraception).

A focus group study was then carried out to explore possible differences between the needs reported in the literature (mainly from Anglo-Saxon countries) and those described by Latinas [20].

### Setting

The study was conducted in the Basque Country, a region in the north of Spain, with a population of 1.2 million. This region has a public health service, free at the point of use, universal and readily accessible, with local centres that provide care for people living in the surrounding geographical area. During pregnancy, women are monitored through alternating appointments with their primary care midwife and gynaecologist, and in the last trimester, they are invited to attend AE sessions run by one of the midwives at their health centre. Normal deliveries are managed by hospital midwives, usually in tertiary hospitals, with mothers staying in for 2 days after the birth. Once they return home, mothers are cared for by their primary care midwife and babies are assigned to a paediatrician and a paediatric nurse.

### Participants

Four groups of women were established taking into account two variables: 1) perinatal time period: pregnancy vs. postpartum until the end of the official period of maternity leave (which, in Spain, is 16 weeks after the birth); and 2) socioeconomic status with two values (moderately high vs. moderately low). They were recruited consecutively by midwives at six public health centres in districts with populations with different socioeconomic characteristics. The socioeconomic classification was made on the basis of data collected in a telephone interview on the level of education, occupation of the woman and her partner, and the residential area, in line with the criteria of the Spanish Society of Epidemiology [21]. Women who were pregnant or had recently given birth were informed about the study and invited to participate by their midwives in routine appointments. Those who expressed an interest in participating were put in touch with the research team [22]. Of the 40 women invited, 30 agreed to take part (Table 1). The group sessions were carried out at the Primary Care Research Unit of Biscay between September and October 2010.

**Table 1:** Distribution of the women by socioeconomic status and period (pregnancy or postpartum). Implementation of the sessions of the four focus groups (G1-G4)

	Period	Socioeconomic level	Date of the meeting	Number of participants	Mean age (yr)	Age range (yr)
G1	Postpartum	High	26 Oct 2010	10	34.1	30-42
G2	Postpartum	Low	3 Nov 2010	6	34.0	32-36
G3	Pregnancy	High	8 Nov 2010	5	32.8	29-36
G4	Pregnancy	Low	11 Nov 2010	9	27.6	24-38

### Procedure

The sessions lasted for 2 h. The topics covered were problems associated with pregnancy, childbirth or breastfeeding; strategies used to address them; and the usefulness of current AE, as well as respects in which it could be improved. Women gave their opinion about their experiences and wishes, with no restrictions being placed on the ideas put forward. Two members of the research team were present in each session. All the sessions were audio recorded, and were subsequently transcribed.

### Analysis

For the analysis, three members of the research team independently read and annotated the scripts to familiarize themselves with the content. The next step was to collate the information related to the various topics discussed. This process requires multiple revisions of the text assigning codes to segments of the text, to group them in general or specific categories, a task that was facilitated by use of ATLAS.ti software. Then, using these categories and subcategories, and links between them, a conceptual structure was built by each of the analysts. Subsequently, the information was shared and again compared with the text for the final analysis. [22] Lastly, to ensure that the findings were internally consistent, these data were triangulated and compared again with the texts [23].

The information collected from the literature reviews, on AE models and promotion of healthy lifestyle habits, and on women’s needs, together with that from focus groups, was given to a group of experts selected for their interest in research and clinical experience in the area of health promotion. It was a multidisciplinary group composed of: two primary care midwives, one hospital midwife, one gynaecologist, one paediatrician, one paediatric nurse, one nurse specialist in breastfeeding, one specialist in health education, one epidemiologist and two psychologists specialized in qualitative research. All those involved were public sector employees with at least 25 years of clinical experience, combined with teaching or research activity. Their extensive professional experience gave them a broad perspective on changes occurring in maternal and infant care. In the Spanish public system, it is usual that professionals only work in primary care or hospitals.

In the meetings of the experts, discussion and consensus techniques were used. Before each meeting, each member of the group received information regarding the objectives of the upcoming meeting together with support material on a topic, and (from after the first session onwards) a summary of the content of the previous session. In the session itself, the topic was briefly presented and each participant put forward their ideas, these being compared and contrasted with those of other participants. Points of agreement and disagreement were identified, the contributions of all participants were comprehensively explored, and finally, the ideas were ranked by voting. The seven sessions were held every couple of months and lasted around 90 min. In the first session, the participants analysed the needs identified in the literature, and the results obtained from focus groups, bearing in mind the characteristics of these groups. In the second and third sessions, they reviewed the theoretical models used in AE and the variables that were considered relevant to the promotion of healthy habits in health education. In the fourth session, they analysed areas to improve given the difference between current AE and women’s needs. In the fifth session, the objectives for new perinatal education were discussed, and in the last two sessions, a proposal was developed for a new model of perinatal education, analysing its feasibility.

## Results

### Theoretical models for antenatal education

In the early models of preparation for childbirth, from the 1950s to the 1970s, the main objective cited was to reduce fears and conditioned pain. Subsequent models have focused on achieving a positive experience of childbirth that was perceived as part of the transition to motherhood (obstetric psychoprophylaxis) and in which fathers were also involved (Bradley method of natural childbirth). More recently, from the 1990s to the present day, efforts have been made to increase women’s leadership skills and empowerment, strengthening coping strategies [15] or “mindfulness”, which would encourage awareness and decision-making during childbirth [24]. The most recent models of maternal education include an emphasis on healthy lifestyles, promotion of self-esteem, good postpartum adaptation and involvement of the family [25, 26]. Further, AE may be focused on empowering women to adopt healthy behaviours in all areas of their lives, in the hope that they will have a positive influence on those around them [27, 28]. In some cases, such as the CenteringPregnancy® model for group prenatal care, AE is offered from the start of pregnancy, and has the objective of reaching the most vulnerable women, in places where AE is not offered for free and is too expensive for some families [29].

In accordance with the trends observed across the studies consulted, the expert group considered that the most useful models were those that dealt with the transition to motherhood, from the start of pregnancy, as they promote a gradual adoption of healthy habits and a sense of control of the situation.

**Fig. 2:**
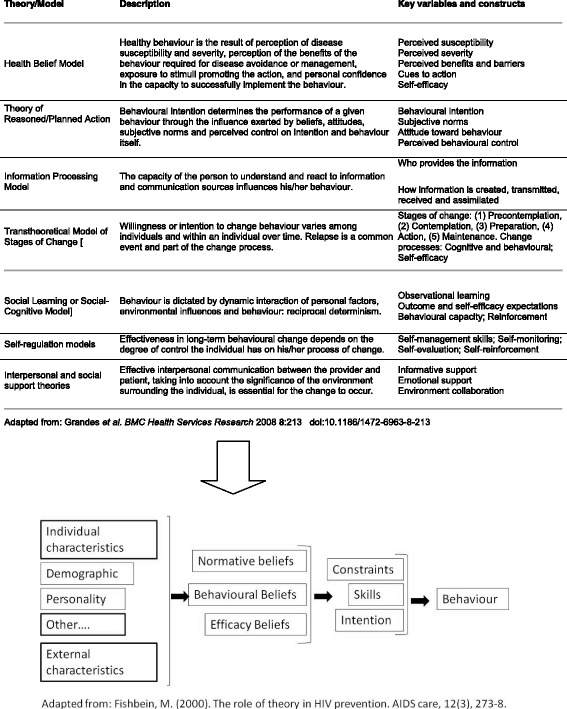
Summary of the main theoretical models of behavioural change in primary care

Figure 2 summarizes some of the models that have been proposed for the promotion of healthy lifestyles. Several of these have been applied to factors relevant to pregnancy and/or childbirth, such as the importance given to a healthy lifestyle [30], belief in the positive impact of behaviour on health [31] and the existence of social support [32]. After identifying these variables as determinants of the acquisition of healthy lifestyle habits, the expert group concluded that an integrative model was needed, one which considered the majority of health determinants and the variables we sought to influence. Specifically, it was decided that the integrative model proposed by Fishbein [33] was the best theoretical model for our purposes, because it considers the intention of individuals as a key determinant of behaviour (together with skills and resources). This intention is influenced by beliefs concerning the harm/benefit ratio of behaviours and how they might be received (degree of acceptance/rejection) in individuals’ social networks, as well as their own capacity to adopt/change the corresponding behaviour. It also considers other variables that may have an indirect influence, such as sociodemographic, cultural, and socioeconomic characteristics, that help strategies to be tailored to specific populations, thereby increasing their effectiveness [33]. If AE really seeks to produce changes in women’s health-related behaviours and experiences, it should take into account all these variables.

### Health needs in women with reproductive age

In the second literature review (as reflected in Fig. 1c), the 21 publications analysed corresponded to: 1 National Institute for Health and Clinical Excellence guideline [34], 5 systematic reviews and 15 other papers, most of which were qualitative and descriptive in nature, with a broad perspective.

The main needs identified were: a) more emotional care to address women’s fears, usually related to a lack of awareness and feeling of being out of control [35, 36]; b) accurate and reliable information [37] acquired through procedural learning, with interaction between participants and flexible timetables [38]; c) specific care for some groups, such as adolescents [39], immigrants [40] or even fathers who request sessions delivered by professionals with similar experiences [41, 42]; and d) an education programme that continues after childbirth [43].

#### Focus groups

The needs expressed by women participating in the focus groups largely coincided with the results from the literature. Women reported fear during pregnancy, for various reasons: concerns about correct development and foetal wellbeing, about childbirth itself, about management and understanding of the newborn, and about the initiation of breastfeeding. In all groups, the women asked for accurate information, flexible schedules and a greater involvement of the fathers.

They wished to have specific information relevant to the period they were going through, rather than far in advance. The women generally had positive views of our health system and of the role of midwives, although they felt a need for more support after the birth and for group activities. Women who had already given birth complained of an excessive pressure to breastfeed and indicated that they found it difficult.

### Areas for improvement in AE

The group of experts identified the following areas for improvement.

1. The objective of the programme should be to accompany women, “helping them to choose” from the available options for birth and child rearing.
2. The programme should ideally be extended to cover the entire process, from seeking to become pregnant until the end of breastfeeding.
3. Flexibility is needed, as a standardized programme, designed by professionals, is useful for some women, but not for increasingly heterogeneous populations with different interests and needs (related to assisted reproduction, multiple pregnancies, ethnic minorities, and pregnancies that are abnormal or at the extremes of maternal age).
4. An AE programme focused only on the woman will fail to take into account other elements that are essential for her wellbeing such as her partner, family and community. These are the source of many norms and customs that have a decisive influence on behaviours such as breastfeeding, child rearing and contraceptive use.
5. Collaboration from external (healthcare and community) resources is necessary to influence the determinants of behaviours.
6. The programme should be subject to a continual improvement process, involving the evaluation of results and most likely regular adaptations.

### Consensus on the needs, models and strategies for a new type of perinatal education

The group reached a consensus on what should be the objective of the new type of programme, defining it as follows:

To *empower* women to take their own decisions for:

- improving their health and that of their family,
- taking responsibility for choosing the type of birth they want and following it through
- using healthcare and non-healthcare resources to promote self-care and preserve and improve their health and that of their families.

A programme of the sort we outline here would include the time before becoming pregnant and would continue at least to the end of the postpartum period, and possibly to the end of breastfeeding (when continued beyond the postpartum period). The professionals and the target population may indicate specific health problems that may be relevant to pregnant women, and these can then be addressed from the recruitment phase, evaluating and guiding women toward specific interventions as appropriate.

**Fig. 3:**
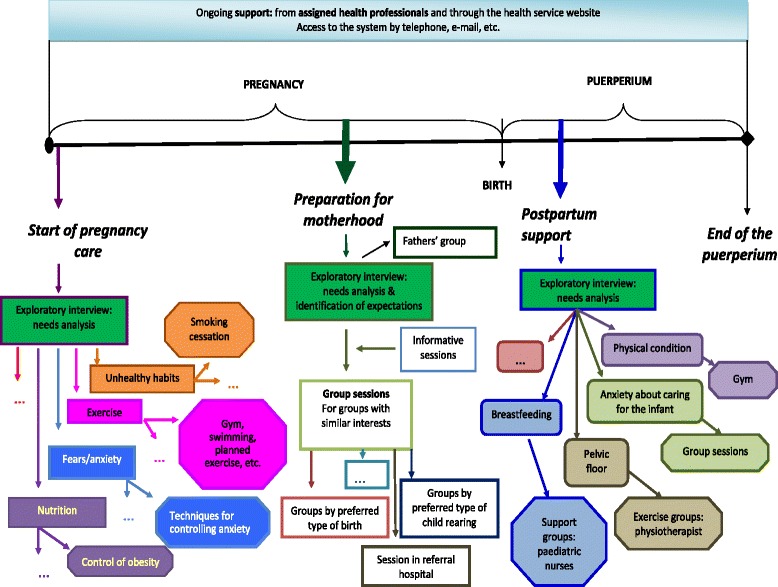
Sequence of possible interventions in the implementation of a perinatal education programme focused on the needs of the woman. Translated from the 2013 Report of the Basque Office for Health Technology Assessment (Osteba)

The preliminary proposal of the group of experts (Fig. 3) includes an exploratory interview when a woman is planning to get pregnant, or at least before week 12 of the pregnancy, to assess lifestyle and management of anxiety, among other issues. Proper identification of any problematic areas, negotiation with the woman, the establishment of adequate channels of communication (with the woman and between professionals) and exploitation of synergies with available resources (health system, community and personal) would together make it possible to work on and evaluate progress towards specific objectives to benefit women through the entire process.

In subsequent consultations, the needs of women are likely to have changed, as they start to focus on the birth. The proposal assumes that there will be different interests depending on personal characteristics (age, ethnicity, number of children), the course of the pregnancy (multiple, abnormal) and the choice of type of birth (from epidural anaesthesia to home birth). The offer would be focused supporting women in their choices and achieving their objectives with respect to the birth.

Immediately after the birth, the needs of women change dramatically. The perinatal education programme should address this issue by setting specific objectives depending on preferences regarding breastfeeding, the existence of perineal damage, starting to use contraception and the need for support from the father/family. At this stage, it is important to draw on personal, and community as well as health system resources.

The midwife seems to be ideal professional to deliver this type of perinatal education programme [44, 45], being seen as the constant, stable point of contact, approachable and accessible to resolve concerns, and responding rapidly to the need for support. However, this type of programme would require the participation of other agents: primary care healthcare and non-healthcare professionals, paediatricians, hospitals, community centres, etc. Hence, there is a need for effective channels for on-going communication between all these agents [46].

## Discussion

The needs of women in terms of pregnancy-related healthcare and childbirth coping strategies are idiosyncratic, they change over the course of the process and they go beyond preparation for childbirth [22]. Models for AE have tried to adapt to this situation and have broadened the focus of the intervention from the birth to include the couple or, in such cases, family that is about to accommodate a new member. However, traditional AE is still mainly being designed by health professionals, focused on women, without consideration of their personal context, limited to the 2 months before childbirth, and with an emphasis on the provision of information. In this context, our findings suggest various areas for improvement including: extending the programme, covering from when women are seeking to become pregnant to the end of the postpartum period or the end of breastfeeding; adopting formats tailored to their lifestyles, which would include the use of information and communication technologies; and accepting that women should take a central role, with the ability to make decisions throughout the entire process of pregnancy, the birth and breastfeeding.

Fortunately, most factors known to positively influence behaviour change are strongly present through pregnancy, birth and child rearing, meaning that this period is an ideal opportunity to promote the general health of mothers, their children and families. Specifically, pregnant women are particularly receptive to healthy lifestyle recommendations, these being perceived as clearly beneficial, if not essential, during pregnancy. Indeed, there is support in society for women adopting healthy behaviours during pregnancy and they themselves often sense that they may regret unhealthy behaviours. In cases when the pregnancy was planned, women tend to have an increased sense of control over their own lives. According to the integrative model of Fishbein [33], modifying intermediate variables, namely, attitudes, beliefs and self-efficacy, will have an impact on behaviour, and hence changes in women may help improve both their health and that of their families. In short, the transition to motherhood is an ideal period for change and, to take full advantage of this opportunity, it is important to offer a perinatal education programme that really meets women’s needs, being comprehensive, flexible and on-going throughout the period, supporting them in taking their own decisions.

There is widespread interest among professionals in developing new AE programmes that are adapted to the needs of today’s women. For example, in Spain, new initiatives have been developed exploiting information and communication technologies and increasing the amount of time dedicated to gym exercises, and breathing and relaxation techniques [47]. In France, it has been proposed that AE should take into account the couple/family and encourage lifestyle changes [1, 44], while many publications from the United Kingdom advocate for a new type of woman-centred AE [10, 20, 48], facilitating the use of women’s preferred strategies [15]. In line with this, in lower income countries, broad educational activities have been recommended, focusing on the woman and with participation of the community, not only of health professionals [49, 50].

The proposal described in this study shares many features with the aforementioned initiatives and recommendations. However, it goes a step further in offering a global framework that includes a comprehensive needs assessment for each woman and allows for the possibility of different programmes tailored to subgroups of women and their context. Further, it stipulates very early intervention, considering this necessary to work on lifestyles, focuses on the birth at the stage when this is seen as a priority by women, and continues with strong support after the birth, seeking to provide new families with skills to adapt to their new situation.

Despite the literature reviews reported here dating from 2010, the current recommendations of the most respected organisations in the field point in the same direction [49–51]. Specifically, in 2016, the US Preventive Services Task Force [52] has recommended that maternal education programs should be extended, throughout the pregnancy and postpartum, and seek to achieve both a healthy pregnancy and a positive transition to maternity, including working on the mother’s self-esteem, skills and autonomy.

This study has limitations typical of an exploratory study, being more a first step towards the redesign of AE than a definitive answer. Regarding the use of focus groups, selection criteria were applied to create homogenous groups in order to maximise rapport and hence interaction between participants; however, this meant that we did not include women with different needs, such as adolescents, extreme socioeconomic groups, or groups of fathers, the latter being especially relevant. On the other hand, given the nature of the approach proposed for the continual improvement of the programme, this having been suggested by people who would be involved in its implementation, the most important characteristics of each AE group would be defined on a case-by-case basis and the programme tailored to the target population.

## Conclusions and practical implications

The development of this framework is to be continued with piloting, as described by the UK Medical Research Council [16]. The interventions seeking to encourage lifestyle changes, promote more participative deliveries, and extend the duration of breastfeeding, for example, should be selected and prioritized by the target population and professionals. It is envisaged that changes will emerge from small teams, influenced by the characteristics of individuals, professionals and the local population [33], and be rapidly piloted in parallel with very small modifications in line with the Plan-Do-Study-Act cycles used by the Institute for Healthcare Improvement [53]. The effectiveness of each pilot study is to be assessed immediately, enabling proposals of micro-teams to be adopted or rejected by others. From the planning stage, we must consider factors that influence the feasibility of the proposed intervention. For this reason, it is necessary to consider interventions at multiple levels, bearing in mind not only the attitude of the midwives and other professionals involved but also the resources available. In relation to this, implementation research indicates factors that determine the applicability of interventions, such as the internal and external context, the type of target population [54] and user perception of the intervention [55, 56]. Evaluation frameworks such as the Reach Effectiveness Adoption Implementation and Maintenance (RE-AIM) model recognise this need to assess both the implementation itself and its effectiveness [57]. With this type of approach, we learn more about the effectiveness of each intervention, but also about the feasibility of delivering it, as well as barriers, facilitating factors and potential outcomes. In this way, we believe that we are making promising progress towards the renewal of perinatal education with a proposal that can be tailored to different populations and settings.

## Abbreviations

AE: Antenatal education
OSTEBA: Basque Office for Health Technology Assessment
RE-AIM: Reach Effectiveness Adoption Implementation and Maintenance
